# Epigenetic Modifications Induced by the Gut Microbiota May Result from What We Eat: Should We Talk about *Precision Diet* in Health and Disease?

**DOI:** 10.3390/metabo13030375

**Published:** 2023-03-02

**Authors:** Katerina Reva, João Laranjinha, Bárbara S. Rocha

**Affiliations:** 1 Center for Neuroscience and Cell Biology, University of Coimbra, 3000-548 Coimbra, Portugal; 2 Faculty of Pharmacy, University of Coimbra, 3000-548 Coimbra, Portugal

**Keywords:** diet, microbiome, microbiota–host interactions, epigenetics, microbiota–epigenetic interactions

## Abstract

Diet is currently considered one of the most important adjustable determinants of human health. The gut microbiota, the collection of microorganisms that inhabit (mainly) the distal bowel, has recently been shown to ensure critical physiological functions, such as immune, metabolic and neuropsychiatric. Many of these biological effects result from the production of bacterial metabolites that may target host cells, tissues and organs. In line with this rationale, epigenetics has brought new insights to our understanding of how environmental factors influence gene expression and, interestingly, gut microbiota metabolites have recently been proposed as novel and significant inducers of epigenetic modifications. Efforts have been dedicated to unveil how the production of specific metabolites influences the activity of epigenetic writers and erasers in order to establish a mechanistic link between gut microbiota, epigenetic modifications and health. Recent data is now evidencing how specific microbial metabolites shape the epigenetic landscape of eukaryotic cells, paving new avenues for innovative therapeutic strategies relying on diet-driven microbiota: epigenetic interactions. Herein is discussed the impact of diet on gut microbiota and the molecular mechanisms underlying microbiota–host interactions, highlighting the influence of diet on microbiota metabolome and how this may induce epigenetic modifications in host cells. Furthermore, it is hypothesized that epigenetics may be a key process transducing the effects of diet on gut microbiota with consequences for health and disease. Accordingly, innovating strategies of disease prevention based on a “precision diet”, a personalized dietary planning according to specific epigenetic targets, are discussed.

## 1. Introduction

The onset and development of diseases is dictated by genetic heritage and environmental triggers. Living in the interface between the external environment and human host cells, gut microbiota is critical to regulate the complex yet delicate interaction between environmental factors and the host [1]. The occurrence of a dysfunctional gut microbiota, i.e., dysbiosis, has been associated with multisystemic diseases including cancer, autoimmune, cardiovascular and metabolic diseases, as well as neuropsychiatric disorders [2,3,4,5]. The mechanisms underlying such effects are not yet clear, but it is becoming increasingly evident that dysbiosis compromises not only the structure of bacteria communities, but also their metabolic activity [1]. In fact, the interkingdom crosstalk between gut microbes and the host is ensured by the production of microbiota-imminent molecules (the so-called pathogen-associated molecular patterns, PAMPs) that bind to receptors expressed by host cells, such as Toll-like receptors (TLRs) and other pattern recognition receptors (PRRs), thereby modulating several pathways on host gastrointestinal mucosa and beyond. In particular, products derived from microbial enzymatic activity, such as short-chain fatty acids (SCFAs), are physiologically relevant molecules with an important immunomodulatory function [6]. Many of these product’s effects occur through epigenetic modifications, i.e., changes in gene expression that are not dictated by the DNA sequence, such as DNA methylation and histone methylation and acetylation, to cite the most studied modifications [7]. The enzymes responsible for epigenetic modifications use several metabolites as substrates, including molecules derived from microbial metabolism which, in turn, may also modulate the activity of such enzymes [8,9,10,11]. For example, SCFAs inhibit histone deacetylases, thereby regulating several physiological pathways, including maintenance of immune homeostasis in the colon [4] or, instead, may trigger pathogenic events leading to the recruitment of neutrophils and aggravated inflammatory responses [12]. Because such effects underly both physiologic and pathologic conditions, the interaction between gut microbiota metabolites and epigenetic enzymes has great potential regarding novel therapeutic approaches. Indeed, over the last years, several studies have shown the success of metabolite-based changes of functional epigenome [1,6], leading to the emergence of a new paradigm in pharmacology: epigenetic drugs [11]. Considering that the metabolic profile of gut microbiota is, in great part, influenced by food, several studies have explored the modulatory effects of diet on gut microbiota richness and diversity and ensued impact on human health [1,6,8,13,14,15]. It should be mentioned that dietary products might, however, have a direct impact on gut microbiota or such an impact might be indirectly promoted via derivatives resulting from preliminary steps by which ingested dietary products suffer chemical/biochemical reactions in the saliva and stomach, of which the nitrate–nitrite–nitric oxide pathway is a prime example [16].

In this review it is proposed that, once an epigenetic target of interest is identified, dietary changes may be used to modulate metabolite production, thereby tuning microbiota–host interactions with epigenetic outcomes useful for health promotion and disease prevention. As a conceptual background for this hypothesis, we provide an overview on the effects that various types of diet have on microbiota structure (populations) and function (metabolome). Metabolic requirements for epigenetic activity are also explored from both a physiologic and pathologic viewpoint, discussing relevant examples in which changes in microbial activity influence epigenetic changes of chromatin. Finally, by identifying possible molecular targets for epigenetic enzymes, we explore the effects of diet in disease prevention and mitigation by means of the diet–microbiota–epigenetics triade.

## 2. Gut Microbiota

All surfaces of the human body are populated by microorganisms. Such organisms live in dynamic communities, consisting of bacteria, viruses, fungi and archaea, collectively known as the microbiota [5]. The human gastrointestinal tract harbors c.a. 70% of the human microbiota, with different composition depending on the anatomic location, exhibiting an increased density from the proximal to the distal gut [5,6]. Gut microbiota is essential for several physiological processes including food digestion, synthesis of bile acids and vitamins, immune response, epithelial barrier function, prevention of pathogen and opportunistic outgrowth and modulation of host gene expression [4,5,15]. The biological functions of the microbiota are not confined to the gastrointestinal mucosa since some PAMPs may be absorbed into the systemic circulation and act as ligands of G protein-coupled receptors (GPCRs), aryl hydrocarbon receptors and pregnane X receptors (PXR), which will, in turn, trigger intracellular signaling cascades, contributing to systemic homeostasis, of which the activation of Treg cells, IgA production and decrease of proinflammatory cytokines are paradigmatic examples [6,15].

Given the intricate communication between the gut microbiota and the environment, this superorganism is susceptible to several factors from both the external environment and host cells. Indeed, various life events and other determinants such as delivery mode, breastfeeding status, age, antibiotics, drug intake and exercise can modulate the human microbiota [15,17]. Of note, many of the influencing factors of the microbiota are environmental and, in this context, diet has also been shown to be a key determinant in shaping the microbial architecture [1,6,18].

### 2.1. The Impact of Diet on Gut Microbiota

In the past few years, several studies have reported the influence that diet, primarily determined by cultural, geographic and socio-economic conditions, has on gut microbiota profile [19,20]. For example, a comparative study showed that Italian children had their microbiota enriched with bacteria from the genera *Bacteroides* and *Alistipes*, whereas the diet of African children lead to enrichment with *Prevotella* and *Xylanibacter* [19]. In line with this, other studies evidenced that non-Westernized populations, which consume mainly raw or wild food, have higher levels of microbial richness and biodiversity than Western populations [20,21]. These studies made clear that among the three primary enterotypes, Bacteroides, Firmicutes and Prevotella [17], a “Westernized” diet, rich in animal protein and saturated fats, results in Bacteroides dominance, whereas a rural, plant-based diet, consisting of high-fiber containing foods, stimulates the abundance of Prevotella [15]. Other dietary styles, such as of a calorie-restrictive regime in overweight adolescents (10 weeks of 10–40% reduction in energy intake) evidenced a decrease in *Clostridium coccoides* and *Bifidobacterium* genus, and increase in *Bacteroides fragilis* [22]. Zimmer et al. reported that compared to omnivores, vegans had significantly lower counts of *Bacteroides* spp., *Bifidobacterium* spp., *Escherichia coli,* and *Enterobacteriaceae* spp., whereas other species such as *Klebsiella* spp., *E.coli biovars, Citrobacter spp.* and *Clostridium* spp. did not differ between the groups [23]. Nutrients can shape microbial growth, inducing both reversible, short- and long-term alterations on microbiota communities [1,18,24]. A detailed description of microbial alterations induced by different dietary patterns is shown in Table 1. Worthy of note, the oscillation of bacterial populations closely reflects the type of ingested nutrients. For example, members of *Bifidobacterium, Bacteroides* and *Ruminococcus* genera express carbohydrate-active enzymes (CAZymes) allowing the hydrolysis of indigestible carbohydrates such as resistant starch, cellulose and fructo-oligosaccharides (FOS) [1]. Thus, carbohydrate-rich diets promote the increase of such genera as a means for an efficient digestion [1,6]. In a similar way, *Bacteroides, Alistipes* and *Bilophila* are tolerant to bile chemical composition and have been shown to increase in response to animal-based diets [18].

Beyond these overall modifications on gut microbiota evoked by chronic consumption of particular dietary products, the fine-tuning of diet-driven microbiota changes is far from being understood in detail. Not only are the dynamic microbiota modifications by acute consumption of products largely unknown but, adding to some controversy among the literature, factors such as circadian rhythm, eating frequency and overnight fasting can also contribute to the oscillation of microbial populations [27]. However, considering that several lines of evidence support a dynamic relationship between diet and gut microbiota profile, the case can be made that by modulating the structure of gut microbial communities and likely their metabolic profile, a precise diet may adjust microbiota–host communication with implications for human physiology.

### 2.2. Microbial Metabolites as Critical Transducers of Microbiota–Host Signaling

Some bacteria strains are specialized in metabolizing specific nutritional components, thereby producing metabolites that are normally not produced by the host [6] (more details are described in Table 2). These metabolites, which include SCFAs, vitamins (vitamin K and B), secondary bile acids (not produced by the host), trimethylamine N-oxide (TMAO) and many others [6,28] are physiologically active molecules that can affect human physiological responses towards health or disease [1,4,6,8,15]. Among the metabolites mentioned in Table 2, SCFAs receive particular attention since they are the main end-products of bacterial fermentation [29] and have been associated with important biological functions, notably (1) providing energetic substrate for intestinal epithelial cells as well as for gut bacteria, (2) modulating metabolic profiles by increasing PGC-1α expression in brown adipose tissue and AMPK activity in liver and muscle, associated to reducing plasma cholesterol, normalizing glucose levels, decreasing fat accumulation in white adipose tissue and increasing fatty acid oxidation, (3) increasing gut mucosal barrier function and immune tolerance, by inhibiting nuclear factor-kB (NF-kB) signaling pathway, activating interleukin-18 (IL-18) production, reducing T-cell activation and increasing colonic regulatory T cells and, finally, (4) stimulating the immune system within the gut and systemically by interacting with several GPCRs, such as GPR41, GPR43 and GPR109A, which will promote an anti-inflammatory cell phenotype [12,29,30].

A critical concept that adds a further layer of complexity on the microbiota–host interaction is that microbial metabolites may exert distinct functions depending on factors such as metabolite concentration and host cells metabolic status, among others. This is the case of butyrate, one of the known SCFAs. Depending on its concentration, butyrate may serve as a source of energy or a tumor-suppressive metabolite. For instance, in normal epithelial cells, butyrate undergoes β-oxidation in the mitochondria, generating FADH2 and NADH which are used in the electron transport chain to ultimately produce ATP [13]. However, in hypermetabolic and highly proliferative cells, such as cancer cells, butyrate accumulates in the nucleus eventually inducing apoptosis [13]. Moreover, butyrate may also inhibit tumorigenesis by binding to GPR109A, a receptor that not only activates a signaling cascade that leads to differentiation of naïve T cells into Treg cells, but also induces production of interleukin-10 (IL-10) and interleukin-18 (IL-18) [12,15].

In addition to SCFAs, other metabolites produced by gut microbiota have been associated with health benefits. To cite just a few, ellagic acid from berries and walnuts has been shown to be metabolized to urolithins with anti-inflammatory and antitumorigenic properties [39] and garlic metabolism originates diallyl disulfide, a potent HDAC inhibitor [28]. Glucosinolate, present in cruciferous vegetables, is metabolized into sulforaphane, an isothiocyanate that turns on anti-cancer genes [9]. Of note, although these vegetables possess myrosinases (a member of glycoside hydrolase family that cleaves a thio-linked glucose, thus a thioglucosidase), after a cooking process at high temperature these enzymes are denatured, leaving sulforaphane production dependent on the microbiota [13]. Other examples will be further explored in detail.

Despite the emerging beneficial effects of metabolites produced by gut bacteria, some reports, both in humans and mice, suggest that the gut microbiota may also be associated with pathogenesis. Indeed, dysbiosis (altered gut microbiota structure and/or function) has been associated with a wide range of disorders, such as obesity [26,41], autoimmune diseases [4,34], diabetes [41], cancer [13,42] and neuropsychiatric diseases [3]. Moreover, causal relationships have been established between dysbiosis and disease and, among these, Parkinson’s disease (PD)—in which a decreased production of butyrate has been consistently associated with symptomatology and pathophysiology in PD patients—and autism spectrum disorder are the best known examples [43].

In this context, not only a depletion of bacteria belonging to families that are producers of beneficial metabolites may occur but, moreover, some dietary components originate metabolic end-products with harmful effects. L-carnitine, found mainly in red meat, choline and phosphatidylcholine are metabolized into trimethylamine N-oxide (TMAO), which acts as a proatherogenic compound [32]. Several studies have shown that TMAO induces both endothelial and intima inflammation, with the production of reactive nitrogen species and, as consequence, LDL oxidation [32,34] and platelet aggregation leading to increased risk of thrombosis [35]. In addition, TMAO has also been implicated in the pathogenesis of chronic kidney disease and type-II diabetes again by generating an oxidative environment [34,44]. Although TMAO is a prime example of a microbiota-derived toxin, several other metabolites are known to induce detrimental effects. Tryptophan-derived indole is an uremic toxin associated with chronic kidney disease [34,44] while tyrosine-derived 4-hydroxyphenylacetic acid (4EPS) is metabolized in the liver into *p*-cresol and *p-*cresylsulfate, which are also associated with uremic syndrome [34]. Moreover, phenylalanine-derived phenylacetic acid has been associated with liver inflammation and, ultimately, with the development of non-alcoholic steatohepatitis [38]. Of note, most of the mentioned mother compounds, such as tryptophan, tyrosine and phenylalanine are commonly ingested amino acids found in protein-rich foods such as meat, fish, eggs, beans, cheese and nuts [36].

**Figure 1:**
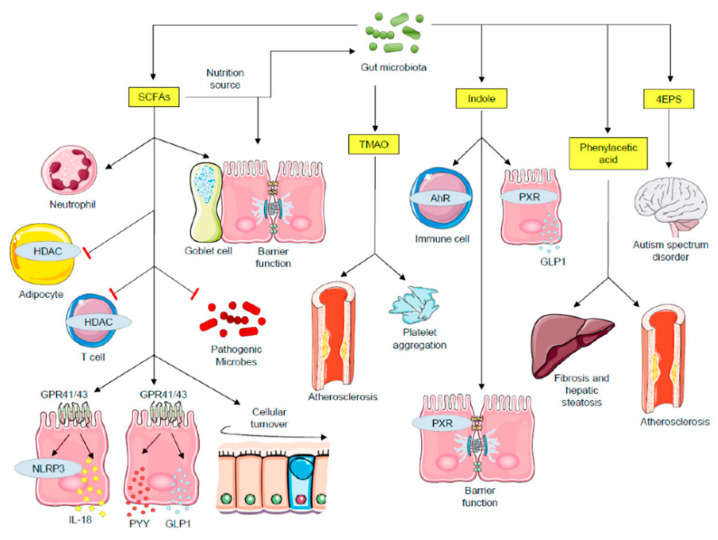
Impact of microbial metabolites on host physiological mechanisms. SCFAs functions include: major nutrition sources for intestinal epithelial cells and gut microbiota; promote barrier function and mucin expression in goblet cells; recruit neutrophils; inhibit pathogenic microbe proliferation by creating an acidic pH condition; SCFAs inhibit HDACs both in adipocytes and in T cells. Whereas HDAC inhibition in adipocytes triggers apoptosis, in T cells it stimulates cell activation. SCFAs activate GPR41 and GPR43, leading to NLRP3 activity and secretion of IL-18, PYY and GLP1. Secreted PYY and GLP1 act on the regulation of energy expenditure and food intake in the central nervous system. TMAO triggers cardiovascular diseases and platelet aggregation. Indole functions include: modulating immune responses through AhR signaling; interacting with PXR, leading to barrier function regulation and GLP1 secretion. The 4EPS is believed to lead to autism spectrum disorder, with the inherent mechanisms being explored. Phenylacetic acid induces fibrosis and hepatic steatosis, contributing to the development nonalcoholic steatohepatitis (NASH). AhR—aryl hydrocarbon receptor; GLP1—glucagon-like peptide 1; GPCRs—G protein-coupled receptors; HDAC—histone deacetylases; PXR—pregnane X receptor; PYY—peptide tyrosine-tyrosine; SCFAs—short-chain fatty acids; TMAO—trimethylamine N-oxide; 4EPS—4-ethylphenylsulfate.

From the above mentioned evidence, it is clear that diet appears as a powerful tool to modulate host physiological responses and an instrumental conclusion can be drawn that depending on gut microbiota composition, different diet-derived metabolites may be detected in the plasma and, therefore, different physiological outcomes are expected to occur depending on this circulating profile. The underlying molecular mechanisms for this complex diet–microbiota interaction with implications for host biological responses is one of the big future research challenges, but it is of note that recent data suggests that epigenetic modifications may play a significant role [8,14,33,45,46]. In view of this promising new trend, the putative mechanisms through which diet shapes gut microbiota profile and therefore microbial metabolome, with consequent epigenetic modifications in host cells, need to be addressed (Figure 1).

This image was designed using free scientific images from Shutterstock and Servier Medical Art collection.

### 2.3. Epigenetics and Epigenetic Modifications in Disease

Epigenetics, a route by which the environment might interact with the human genome, has recently moved to the epicenter of modern medicine and, as such, targeting the epigenome has been proposed as a novel approach to prevent disease and develop new therapies [7]. Epigenetics refers to dynamic and heritable chemical changes that occur both on DNA, non-coding RNAs and core histones, influencing gene expression without altering the DNA sequence [7,47]. The most studied epigenetic changes include post-translational modification (PTM) of amino acid residues (located on both histone core and tails) by acetylation and methylation as well as DNA cytosine methylation [7]. The collection of known PTMs is large and includes biotinylation, phosphorylation, sumoylation, ubiquitination, ADPribosylation, among others [48]. Although known to occur, the functional impact of these modifications is yet to be studied in detail. Epigenetic modifications can be associated either with genetic activation or repression, depending on the type of PTM, the targeted molecule (DNA or histone amino acid residue), as well as the specific location of DNA nucleotides and amino acid residues, and degree of modification [49]. For example, histone H3 lysine 4 methylation (H3K4me) is a gene-activating PTM [49], while histone H3 lysine 9 trimethylation (H3K9me3) is associated with gene repression [50]. An innovative and remarkable concept is that epigenomic modifications and gene expression are influenced by metabolism and several environmental factors, such as diet, exposure to toxins and pollutants, smoking and physical activity [51]. In line with this rationale, gut microbiota metabolites may establish the link between the effects of diet both on gut microbiota structure and function and the modification of host genome [25,28,33]. Interestingly, several lines of research now suggest that aberrant epigenetic activity may degenerate in disease, but some gut microbiota metabolites have been shown to prevent or mitigate these responses.

Given that epigenetic pathways are crucial for normal biological functions, it is not surprising that epigenetic dysregulation plays a significant role in pathological processes. Accordingly, studies associate aberrant epigenetic modifications to diseases such as cancer [11,42,52], rheumatoid arthritis, inflammatory bowel disease, metabolic syndrome, type 1 and type 2 diabetes mellitus [2,4,10,41] and schizophrenia [3]. Mechanistically, several epigenetic modifications may lead to dysfunctional cell signalling and, among these, the most well understood are aberrant DNA methylation patterns at CpG promoter regions that modulate the access of transcription machinery to DNA [11,53]. For instance, global DNA hypomethylation is known to activate growth-promoting genes in tumors, including cyclin D2 in gastric cancer and carbonic anhydrase IX in renal cell cancer [53].

The epigenetic chemical notation is reversible and catalysed by enzyme families that induce (the “*writers*”) and remove (the “*erasers*”) the chemical groups from DNA or the histones. The regulation of these enzymatic activities is, thus, essential for the biological outcome of the herein proposed diet–microbiota–epigenetic axis as, although necessary to induce epigenetic modifications under physiological conditions, it may also trigger aberrant modifications with deleterious consequences [54]. For instance, under a scenario of uncontrolled cell proliferation, several alterations on the enzymatic activity of “writers” and “erasers” have been reported: (1) DNA methyltransferases (DNMT) 1, 3a and 3b are overexpressed, leading to increased DNA methylation of promoter regions of tumor suppressor genes [55], (2) histone deacetylases (HDAC), such as HDAC1, HDAC2, HDAC3 are overexpressed, leading to epigenetic repression of tumor suppressor genes such as *CDKN1A* [56], (3) Set2 methyltransferases (a family of histone methylation enzymes) are both mutated and overexpressed, leading to hypermethylation of H3K36 which is associated with cancer progression [57] and (4) SIRT6 (sirtuin family of proteins responsible for histone deacetylation) is depleted [58]. This aberrant activity is observed not only in cancer but also in other diseases. For more examples of diseases associated with dysfunctional epigenetic mechanisms, see Table 3.

The association between deregulated enzymatic activity and disease leads to the tangible hypothesis that by modulating the activity of the enzymes involved in the dynamic modifications of chromatin (the “writers” and the “erasers”), such as DNMT and HDAC, it is possible to prevent, mitigate or even revert pathogenic mechanisms. Thus, the concept of epigenetic drugs, that aim to regulate the activity of these enzymes, has emerged as a novel therapeutic approach. For instance, HDAC (targeting classes I, II and IV) and DNMT inhibitors have been proposed as anticancer agents [54,56]. Similarly, the activation of sirtuins (SIRT1 and SIRT3) has been shown to promote insulin secretion, inhibit adipogenesis, decrease fat storage and enhance lipid utilization in the muscle, improving the prognosis of diseases such as type 2 diabetes, obesity and neurodegenerative diseases [61].

### 2.4. Microbiota Metabolites Modulate the Activity of the Enzymes Involved in Dynamic Chromatin Modifications

**Figure 2:**
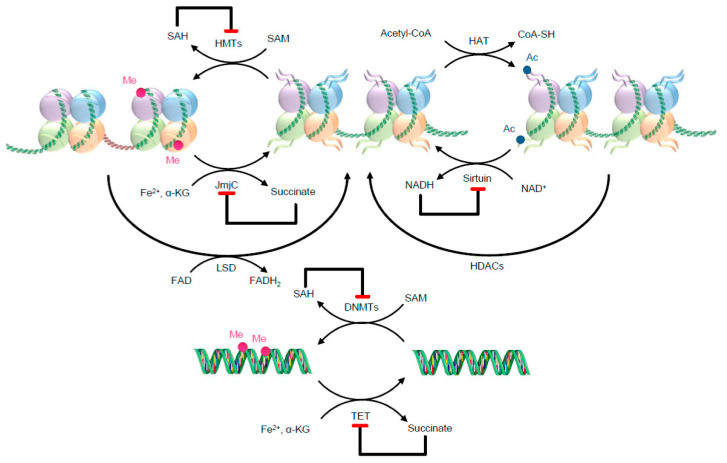
Examples of metabolic needs for two well-known epigenetic writers and erasers. The metabolites might work as substrates, allosteric modulators, and co-factors. Red ending arrows identify inhibitory effects. As illustrative examples, histone acetyltransferases (HATs) use acetyl-CoA as a substrate for histone acetylation, transferring the acetyl group to lysine residues of histones. For the methylation reaction, DNMTs and HMTs use SAM [63]. After the methylation reaction, SAM turns into its reduced form SAH, which potently inhibits DNMTs and HMTs. HDACs are separated into classes that use NAD^+^ for their deacetylation activity, such as class III enzymes (also called sirtuins), and zinc-dependent classes, such as class I, II and IV, although here only sirtuins are illustrated. After the deacetylation reaction NADH (reduced form of NAD^+^) is formed, which inhibits sirtuin activity. Ac—Acetyl; DNMTs—DNA methyltransferases; FAD—flavin adenine dinucleotide; FADH_2_—reduced flavin adenine dinucleotide; HATs—histone acetyltransferases; HDACs—histone deacetylases; HMTs—histone methyltransferases; JmjC—JumonjiC; LSD—lysine-specific demethylases; NADH—reduced nicotinamide adenine dinucleotide; NAD^+^—oxidized nicotinamide adenine dinucleotide; Me—Methyl; SAH—S-adenosyl-homocysteine; SAM—S-adenosylmethionine; TET—ten-eleven translocation methylcytosine dioxygenase family; α-KG—α-ketoglutarate.

A particular interesting notion regarding epigenetic modifications is that metabolism, by providing intermediary metabolites as substrates to the enzymes involved in the dynamic modification of chromatin, modulates the epigenetic signature. Thus, the regulation of the transcriptional networks that imparts a specific cell in a specific organ its physiological identity is connected to its metabolism that, in turn, is influenced by environmental factors, including the diet [62]. Among these metabolites, adenosine triphosphate (ATP), acetyl-coenzyme A (acetyl-CoA), flavin adenine dinucleotide (FAD), oxidized nicotinamide adenine dinucleotide (NAD^+^), S-adenosylmethionine [63], succinate, fumarate and α-ketoglutarate (α-KG) can either enhance or reduce the activity of the enzymes as illustrated in Figure 2 [10,33,63]. *For the purpose of the hypothesis herein described, it is noteworthy that, in addition to host metabolites, microbial metabolites may also interfere with epigenetic modifications* [25,45,46]. These can act as inductors, participants or modifiers of epigenetic enzymatic activity, thereby contributing as one of the mechanisms through which gut microbiota mediates the impact of diet on host homeostasis.

This image was designed using free scientific images from Shutterstock and Servier Medical Art collection.

The metabolic requirements and the interaction of epigenetic enzymes with gut metabolites will be addressed by providing representative examples.

Histone acetyltransferases (HATs) use acetyl-CoA as a substrate for histone acetylation. Hence, acetyl-CoA availability determines histone acetylation, a reaction generally associated with chromatin loosening, consequent increased accessibility, and active transcription [54]. Gut microbiota is considered an important donor of acetyl groups. For example, butyrate, a specific type of SCFA produced from fiber-rich diets through microbial fermentation, can lead to the formation of a metabolic intermediate, butyryl-CoA that, in turn, is a donor of butyryl groups that can be transferred to histones, modifying them. However, it also functions as an HDAC has at low concentrations, stimulating HATs activity and colonocytes growth and proliferation [13]. However, at high doses (mM), butyrate has deleterious effects to the cell, a mechanism that will be further detailed. Besides butyrate, acetate and propionate are also converted to acetyl-CoA, serving as energy sources in the liver, and, indirectly via acetyl-CoA, as substrates for HATs [14].

Histone deacetylases (HDACs) are classified into NAD^+^-dependent class III enzymes (also called sirtuins), and zinc-dependent classes, such as class I, II and IV [28]. Sirtuins are signaling proteins that use NAD^+^ as co-substrate to remove covalently attached acetyl groups [11]. The NAD^+^/NADH *ratio* derived from deacetylation reactions plays a central role in regulating cellular energy metabolism [11,61]. Low NAD^+^ concentration and a consequent decreased NAD^+^/NADH *ratio* have an inhibitory effect on sirtuins activity [11]. On the contrary, after caloric restriction (20–40%), there is an increase of the NAD^+^/NADH *ratio*, enhancing sirtuin activity [61]. Also, a low NAD^+^/NADH *ratio* can be observed in cells with high glycolytic activity, such as cancer cells, increasing the production of acetyl-CoA [11]. These metabolic alterations promote the activity of HATs which, combined with decreased activity of sirtuins, contribute to histone acetylation and, ultimately, to altered gene transcription [11]. Regarding interactions between microbial metabolites and HDACs, butyrate and sulforaphane, which have antiproliferative properties, stand out as HDAC inhibitors [9,10,13].

DNA methyltransferases (DNMTs) and histone methyltransferases (HMTs) use SAM as a methyl donor group [42], converting this compound into S-adenosyl-homocysteine [11,64], which in turn potently inhibits DNMTs and HMTs. Hence, SAM/SAH ratio influences methyltransferase activity [11] and an excessive SAM availability can contribute to CpG hypermethylation, which inappropriately silences genes, such as tumor suppressor genes RASSF1 and SOCS2 [11]. The most important sources of SAM are folate [25,28,46] and other complex B vitamins such as B2, B6 and B12 [25,28,46]. These are obtained both from foods such as green leafy and cruciferous vegetables as well as nuts [46] and from microbial synthesis, in which *p-*aminobenzoic acid (pABA) and pteridine precursors (DHPP) are used as substrates. Accordingly, *Bifidobacterium* and *Lactobacillus* species have been shown to synthetize significant amounts of folate [33] and sulforaphane, formed after microbial metabolization of garlic and cruciferous vegetables, has been shown to have several anticancer effects, downregulating DNMTs activity in prostate cancer [65].

Ten-eleven translocation family of enzymes [66,67] are dioxygenases that depend on α-ketoglutarate and Fe^2+^ to demethylate cytosine residues from DNA [11]. Given the structural similarity with α-ketoglutarate, compounds such as fumarate and succinate, both synthesized by gut microbes, may inhibit α-ketoglutarate-dependent dioxygenases, acting as competitive inhibitors for TETs. Consequently, there is an increase in DNA and histone methylation [11].

*Taken together, these examples support the notion that knowing the interaction between specific PAMPs and enzymes involved in the epigenetic modification of chromatin, opens a new pathway for selecting diets that contribute to increase the bacterial populations, producing the desired metabolites. This hypothesis may be envisaged as a means by which a “precision diet”, paralleling the notion of “precision medicine”, may modulate epigenetic modifications induced by gut microbiota with putative health effects.*

## 3. The Therapeutic Potential of the Diet–Microbiota–Epigenetics Triade

As discussed before, it is herein hypothesized that epigenetics may be a key process transducing the effects of diet on gut microbiota structure and functional capacity, with consequences for health and disease.

Given this conceptual background, it seems plausible that selectively modulating the components of the diet may constitute a novel therapeutic and health-promoting strategy. Of note, over the last decades, several studies have supported the dynamic relationship between diet, microbiota and epigenetics [8,13,25,28,42,46]. In this context, several molecular mechanisms underlying the effect of different types of diet on health outputs have already been forwarded [1,5,68,69,70] and a special focus has been put on “Mediterranean” vs. “Western” diets. The Western diet, which is commonly known to predispose regular consumers to cardiovascular and metabolic complications [71], is rich in saturated and trans fats, sugar, salt and animal protein and low in fibre, fresh fruit and vegetables, mono and polyunsaturated fats [71]. In terms of the impact on microbiota structure, such ingestion pattern is known to increase *Bacteroides, Alistipes, Bilophila* and *Enterobacteria* genera and decrease *Bifidobacterium, Bacteroidetes, Eubacterium* and *Lactobacillus species* [5,18]. These increased microbial populations are known to produce metabolites with deleterious health effects, such as TMAO and indole, and decrease the availability of other metabolites associated with beneficial health outcomes, such as SCFAs and sulforaphane [1]. Consequently, a Westernized diet is associated with an increased risk of non-alcoholic fatty liver disease, cancer, cardiovascular disease, type 2 diabetes mellitus, inflammation and obesity [1,9,68]. On the other hand, a Mediterranean Diet is characterized by the consumption of a broad variety of foods which may, at least in part, justify its health benefits. The Mediterranean diet is rich in monounsaturated and polyunsaturated fatty acids, fiber, polyphenols, protein from vegetables, fresh fruits and vegetables. The intake of fish, poultry and red wine is moderated. The consumption of products such as milk derivates, saturated fats, red and processed meat as well as sweets are rather low [5,69]. Subjects complying to this type of diet exhibit increased populations of *Bifidobacteria, Lactobacillus, Eubacteria* and *Prevotella* and decreased *Clostridium* [1,5,70]. Besides producing metabolites of interest, such as SCFAs and sulforaphane, these bacteria populations show themselves beneficial for producing other molecules with impact on the host homeostasis, including polysaccharide A that boosts immune responses, microbial anti-inflammatory molecule (MAM) that inhibits NF-kB activation and flagellin, a protein component of bacterial flagella that induce acquired immune responses via interaction with Toll-like receptors and has been shown to interact with long non-coding RNA, an RNA molecule that plays important roles in the epigenetic process [5,31]. As a corollary of these beneficial effects, the Mediterranean diet is associated with lower rates of cardiovascular disease, cancer incidence and overall mortality [68,69]. It should be noted that most food components that integrate the Mediterranean diet include compounds such as arabino-oligosaccharides (AOS), fructo-oligosaccharides (FOS), galacto-oligosaccharides (GOS), xylooligosaccharides (XOS), fructans, polydextrose, among others. These compounds are sources of prebiotics—substances that stimulate the development or activity of health-promoting bacteria—contributing for the beneficial health effects of this diet [1,5].

Regarding the epigenetic effects of metabolites produced upon the consumption of specific foods, some of them ought to be explored in detail. These include SCFAs, polyphenols, monounsaturated and polyunsaturated fatty acids, TMAO and uremic toxins.

**Short-chain fatty acids** have been shown to modulate immune response, nourish intestinal epithelial cells and gut microbiota as well as to regulate metabolic pathways [29]. These small molecules are produced by *Faecalibacterium prausnitzii* genera, whose population increases with specific nutritional behavior, including the consumption of proteins from plant origin, unsaturated fats, fiber and polyphenols [5,6]. Furthermore, as discussed above, several mechanisms have been described as underlying the health benefits of SCFAs produced by gut microbiota and the modulation of gene expression via epigenetic mechanisms is precisely one of these. In fact, HDAC inhibition by butyrate has been widely studied given its antiproliferative properties and, thus, potential application in cancer [8,13,15]. Briefly, while in normal cells butyrate is metabolized through β-oxidation in the mitochondria, this pathway is compromised in cancer cells. Although cancer cells may find themselves in the presence of oxygen enough to support oxidative phosphorylation, these tend to metabolize glucose anaerobically, generating ATP in a relatively inefficient manner, through the so-called “aerobic glycolysis” [13]. Under such conditions, butyrate accumulates in the nucleus where it inhibits HDAC activity [72]. This mechanism acquires further relevance since HDACs are overexpressed in cancer cells, where they silence important tumor suppressor genes [11]. Thus, HDAC inhibition by butyrate upregulates genes such as *Fas* and *p21*, significantly preventing cancer cell proliferation and inducing apoptosis [13]. HDAC inhibition by butyrate has also been associated with anti-inflammatory effects [28].

Similarly to butyrate, **sulforaphane** has chemoprotective properties due to its HDAC inhibitory activity [8,13]. In addition, sulforaphane normalizes DNA methylation and activates miR-140 expression controlling tumor growth [51]. The endogenous production of sulforaphane from cooked foods containing its precursors (such as glucosinolates) depends on the activity of certain bacterial species from gut microbiota, namely *Bacteroides thetaiotaomicron, Enterococcus faecalis and Enterococcus faeciu* [8]. The consumption of cooked broccoli or cabbage by an individual with a gut microbiota phenotype poor in these bacterial strains may elicit lower yields of sulforaphane and the protective effects described above are mitigated [8].

**Polyphenols**, secondary plant metabolites, are known to have several beneficial effects regarding human health [1,39,51,73]. However, it is nowadays clear that in view of their limited bioavailability, the health beneficial effects are conveyed by indirect mechanisms [74], including their metabolization by microbiota [75]. That is, dietary polyphenols, are metabolized by gut microbiota yielding physiologically active metabolites. This has been an area of intense research and several robustly-supported examples can be forwarded. Epigallocatechin-3-gallate (EGCG), which is found in green tea, is metabolized to epigallocatechin (EGC) and gallic acid (GA), both of which are HAT inhibitors [8]. Ellagitannins, present in pomegranate, berries, almonds and walnuts, undergo acid hydrolysis in the stomach and small intestine being converted into ellagic acid which in turn is further metabolized by the gut microbiota into urolithins, including urolithin A, B, C and DD [8,39]. Studies suggest that bacteria from the Actinobacteria phylum, Clostridium coccoides group and Lactobacillus genus are involved in urolithin production [8,46]. Urolithins, apparently, exert a wide range of biological effects, including anti-inflammatory, antioxidant, antiproliferative and antiestrogenic [8,46]. Urolithin A is mainly responsible for these effects and it is capable of inhibiting NF-ĸB and mitogen-activated protein kinase (MAPK), downregulating the expression of COX-2 and mPGES-1 under inflammatory conditions [76]. Moreover, urolithin A has been shown to exert antiproliferative effects by increasing pro-apoptotic protein expression (p53 and p21) and inhibiting anti-apoptotic protein expression (Bcl-2) while inducing the production of oxygen reactive species in colorectal cancer cells [77]. In addition, ellagic acid and its derived urolithins, reduce HATs activity in an in vitro inflammation model of tumor necrosis factor (TNF)-stimulated monocytes [8].

**Monounsaturated fatty acids (MUFAs)** and **polyunsaturated fatty acids (PUFAs)** are not produced by bacteria but rather obtained from dietary sources. The consumption of olives and olive oil, rich in MUFAs, is associated with an increased activity of beneficial bacteria such as *Bifidobacterium* and *Lactobacillus* [71]. These bacteria are important producers of SCFAs, including butyrate, but are also responsible for decreasing the production of pro-inflammatory molecules (TNF-α, LDLc, IL-17A) and to protect colonocytes against oxidizing species in mouse models [71]. On the other hand, PUFAs, particularly omega-3 and omega-6 PUFAs, are essential fatty acids that should be obtained from diet and that can be found in fish, nuts and seeds, vegetable and fish oils and soy derivates [71]. Data from biochemical, cellular, animal, epidemiological, and other human studies support omega-3 PUFAs, which include molecules such as α-linolenic acid, as beneficial nutrients on cardiovascular and inflammatory diseases as well as chemoprotective agents [46,78]. In addition, these have been shown to target epigenetic mechanisms, such as histone methylation and miRNA expression [46]. Moreover, omega-3 PUFAs stimulate the growth of LPS-suppressing bacteria, such as *Bifidobacteria* and decrease the abundance of LPS-producing bacteria, such as *Enterobacteria*, suggesting that omega-3 PUFAs, by promoting the microbiota to a healthier condition, might play a beneficial role in inflammation-related conditions such as, for instance, inflammatory bowel diseases [71,78]. Linoleic and arachidonic acids are omega-6 PUFAs and the latter is an important precursor of eicosanoids, including pro-inflammatory prostaglandins [71]. In this context, it is noteworthy that a recent work has supported that *Lactobacillus*-colonized gut microbiota converted the omega-6 PUFA linoleic acid in a hydroxy metabolite, reducing the conversion in the inflammatory eicosanoids cascade [66,71].

Although the health benefits of gut microbiota are now clear, it is also widely accepted that microbiota might be Janus-faced. Indeed, gut bacteria may produce metabolites with detrimental effects on host homeostasis and one such example is TMAO, a metabolite produced by gut bacteria of individuals enjoying an omnivorous diet and whose concentration increases with the consumption of red meat [1,32]. TMAO precursors include betaine, carnitine, choline, crotonobetaine and phosphatidylcholine [34] and it is synthetized by a first step which includes microbial metabolization into trimethylamine followed by hepatic oxidation into TMAO [32]. From a mechanistic viewpoint, TMAO activates both MAPK and NF-kB signaling pathways in endothelial and vascular smooth muscle cells, inducing the expression of genes encoding pro-inflammatory proteins, such as macrophage inflammatory protein 2 and monocyte chemotactic protein, as well as promoting leucocyte adhesion to the endothelium [79]. The detrimental inflammatory actions of TMAO further includes the induction of NLRP3 inflammasome formation and activation, thereby contributing to endothelial injury and promoting atherogenesis [34]. Regarding thrombosis risk, TMAO increases calcium release by the endoplasmic reticulum in platelets promoting aggregation [34,35]. Interestingly, over the last years, research has focused on strategies to constrain microbial synthesis of trimethylamine as a strategy to reduce the risk of thrombosis and cardiovascular disease [37,80]. Some of these approaches rely precisely on the exposure to foods rich in nutrients such as resveratrol, a molecule that inhibits gut microbial trimethylamine production by remodelling gut microbiota [80].

Uremic toxins are compounds excreted by kidney and present in increased concentrations in body fluids and tissues in patients with renal failure and stand out due to their detrimental effects on cardiac and renal health [34,44]. Uremic toxins are produced by fermentation of amino acids such as phenylalanine, tryptophan and tyrosine in the gut (*Proteobacteria* and members of *Firmicutes* family), as well as in the liver after amino acid absorption [34]. Amongst several uremic toxins, indoxyl sulfate and *p*-cresyl sulfate are worth mentioning due to their association with a wide spectrum of adverse outcomes. The first is among the most representative gut-derived uremic toxins which enhances platelet activity, promotes vascular smooth muscle cell calcification, activates NF-kB signaling pathway and inhibits nitric oxide production, increasing the risk of acute cardiovascular events [34,75]. On the other hand, *p*-cresyl sulfate is associated with the production of oxidizing molecules and increased expression of TNF-α, intercellular adhesion molecule-1, and monocyte chemotactic protein-1 in endothelial cells, contributing to plaque formation [34].

## 4. Conclusions and Future Directions

Gut microbiota is in close contact with both the environment and the host, mediating the molecular crosstalk between external agents, the gastrointestinal mucosa and beyond. Considering that gut microbiota can be shaped by dietary patterns, it follows that diet-induced changes are translated to the host by sending messages through specific microbiota metabolites that modulate metabolic and physiological pathways. Worthy of note, part of these gut-derived signals may trigger epigenetic modifications, resulting in gene expression patterns that might impact in physiology and pathophysiology. Several lines of research now show that diet may modulate the structure of gut bacteria communities and their metabolic profile with epigenetic implications, but the underlying mechanisms remain largely unclear. In this regard, it is becoming evident that many pathological states have inherent epigenetic causes, external factors, and that gut microbiota metabolites and dietary behaviors can impact on the progression of disease. Only recently, a few specific mechanisms and pathways have been unveiled; however, in view of the complexity and multitude of individual factors influencing both microbiota–host interactions and the epigenetic regulation of gene expression, a comprehensive understanding of the diet–gut microbiota–epigenetic axis is still largely unclear. For instance, murine models are usually the experimental model of choice but there are phylogenetic differences between rodents and humans that cannot be ignored, including gut microbiota composition. Additionally, one obvious limitation while attributing one specific epigenetic modification to a particular molecule is that nutrients are rarely consumed in isolation and manipulating specific nutrients invariably change the intake of others. A common example comes with high-fat diets that are low in fiber intake and it is hypothesized that the effects on microbial populations are due to low fiber content rather than the elevated fat intake.

In essence, more studies are required in humans, both to widen the knowledge of how microbial populations are shaped according to selective dietary patterns, and to have a better understanding of the food components that work as direct triggers of the diet–microbiota–epigenetics axis. Nevertheless, as discussed here, several lines of research support the notion *that epigenetics may be a key process transducing the effects of diet on gut microbiota structure and functional capacity, with consequences for health and disease.* In association with the assignment epigenetic signatures to a given disease, this knowledge will establish the conceptual background that opens the possibility to manage epigenetic activity via a properly designed diet, herein proposed as a “precision diet”.

## Figures and Tables

**Table 1:** Impact of different types of diet on gut microbiota structure.

Nutritional Behavior	Microbiotic Structure	References
Omnivorous	↑ *Bifidobacterium and Bacteroides* species, *Escherichia coli*, and Enterobacteriaceae.	[6,15]
Vegans/ vegetarian	↑ *Coliforms* (vegan). ↑ *Prevotella* (vegetarian). ↓ *Bacteroides, Bifidobacteria, Escherichia coli and* Enterobacteriaceae.	[23,25]
Diet high in animal protein (temporary)	↑ *Bacteroides, Alistipes, Bilophila* and *Clostridia*. ↓ Firmutes (*Eubacterium rectale, Ruminococcus bromii* and Roseburia species) and *Bifidobacterium.*	[1,5,6,26]
Diet high in plant protein (temporary)	↑ Bifidobacteria and commensal Lactobacilli; ↓ *Bacteroides* and *Clostridium perfringens.*	[5,6]
Diet high in resistant starch (temporary)	↑ Proportions of *Firmicutes* bacteria related to *Ruminococcus bromii*.	[15]
Diet rich in unsaturated fat	↑ Lactobacillus, Streptococcus, Bifidobacteria and *Akkermansia muciniphila.*	[5]
Diet rich in saturated fat	↑ *Bacteroides, Bilophila, Faecalibacterium prausnitzii.*	[5]
Diet high in fiber	↑ Bacterial abundance; ↑ *Bacteroides*, *Bifidobacterium*, *Lactobacilli*, *Roseburia, Eubacteria* and *Ruminococcus* ↓ *Enterococcus* and *Clostridium* species	[5,6,26]
Diet rich in polyphenols	↑ Bifidobacteria and Lactobacilli; ↓ Bacteroides, Clostridia, *Salmonella typhimurium* and *Staphylococcus aureus.*	[5]
Rural diet	↑ *Bacteroidetes* (including the genera Xylanibacter and Prevotella); ↑ microbiotic variety.	[1,6,8]
Urbanized diet	Loss of *Treponema* species; loss of microbiota diversity.	[1,6]
Temporary calorie- restrictive diet	↓ *Blautia coccoides*. ↑ *Bacteroides*.	[1]

**Table 2:** Gut microbiota metabolites, metabolite-producing bacteria and respective dietary substrate (the examples were selected taking into consideration the putative implications for host epigenome).

Gut Microbiota Metabolite	Metabolite Producing Bacteria	Dietary Component of Origin	References
Short-chain fatty acids (SCFAs)	*Faecalibacterium prausnitzii*, *Bifidobacterium* spp.*, Lactobacillus* spp.*, Roseburia* spp.*, Eubacterium hallii.*	Dietary fiber (undigested complex carbohydrates)	[4,5,6,8,14,31]
Trimethylamine N- oxide (TMAO)	*Proteus mirabillis*. Bacteria present in higher abundances in omnivores populations.	L-carnitine, choline and phosphatidylcholine (particularly from red meat)	[1,32,33,34,35]
Indole and indole derivates	*Escherichia coli, Clostridium* spp. and *Bacteroides* spp. *Enterococcus faecalis.*	Tryptophan	[6,34,36,37]
4-hydroxyphenyl- acetic acid and 4–ethylphenyl- sulfate (4EPS)	Species largely unknown.	Tyrosine	[6,34]
Phenylacetic acid	Species largely unknown.	Phenylalanine	[6,34,38]
Isothiocyanate	*Escherichia coli, Bifidobacterium* sp.*, Bacteroides thetaiotaomicron, Enterococcus faecium, Enterococcus faecalis and Peptostreptococcus* sp.	Glucosinolates	[8]
Methylated catechins, phenolic products, and ring fission products (ex. valerolactone)	Species largely unknown.	Catechins	[8,39]
Urolithin	*C. coccoides, Bifidobacterium* spp.*,* and *Lactobacillus* spp.	Ellagic acid	[8,13,39]
Dihydrogenistein, dihydrodaidzein, equol, enterolactone, enterodiol and *O*-desmethylangolensin (*O*-DMA)	*Lactococcus garvieae, Eggerthella* sp. *YY7918, Adlercreutzia equolifaciens, Slackia isoflavoniconvertens, Slackia equolifaciens, Slackia* sp. *NATTS.*	Phytoestrogens	[13,34]
Protocatechuic acid (PCA)	Species largely unknown.	Anthocyanins	[34,39]
Nitrite	Species largely unknown, although studies point out Actinobacteria and Firmicutes as highest nitrate-reducers.	Nitrate	[40]

SCFAs include mostly acetate, butyrate and propionate (proportion of 3:1:1), and others less frequent, such as caproate, formate, and lactate [8,14]. Indole and 4EPS are further metabolized in the liver, originating respectively, indoxyl sulfate and *P*-cresylsulfate [34]. Nitrite is further metabolized into nitric oxide [40]. Ellagic acid is formed after ellagitannin acid hydrolysis [39].

**Table 3:** Aberrant epigenetic modifications related to disease.

Disease	Epigenetic Modifications	References
Colorectal cancer	Epigenetic modifications targeting several genes, including APC, GATA4, MLH1 and p16INK4a.	[13]
Irritable bowel syndrome	Diminished miR-199 level, correlating with TRPV1 (transient receptor potential cation channel subfamily V member 1) upregulation and increased visceral sensitivity.	[59]
Inflammatory bowel disease	MiRNA dysregulation in Th17 cells, affecting its function and differentiation.	[60]
Obesity	Several obesity-related genes with differential methylation, including CD36, CLDN1, HAND2, HOXC6, SORBS2 and PPARG; H3K9me in white adipose tissue regarding differentiation from white to brown adipose cells.	[41]
Non-alcoholic fatty liver disease (NAFLD)	PNPLA3 (patatin-like phospholipase domain containing 3) gene hypermethylation.	[41]
Lupus erythematosus	Overexpression of various genes in CD4+ T cells, like ITGAL, PRF1, TNFSF7, TNFSF5, leading to the activation of B cells and over production of autoantibodies.	[50]
Rheumatoid arthritis	Patients with RA have lowered DNA methyltransferase expression in Treg cells and significantly reduced DNA methylation in the Foxp3 promoter. There are other interesting findings in the existing studies [50].	[50]
Type 1 diabetes mellitus (T1DM)	Several aberrant DNA and histone modifications. Regarding DNA methylation, examples include increased methylation in the insulin-like growth factor-binding protein 1 (IGFBP1) gene, leading to increased levels of circulating IGFBP1; hypermethylation of promoter regions of Interleukin-2 receptor alfa chain gene; hypermethylation of Foxp3 gene promoter; etc. An example of histone aberrant epigenetic modifications are HDACs reduced expression in CD4+ T cells.	[50]
Type 2 diabetes mellitus (T2DM)	H3K27me3 modification in myocytes, downregulating genes responsible for muscle function and upregulating genes involved in T2D inflammation. Several genes related to risk of T2D with aberrant DNA methylation, including FTO, KCNQ1, IRS1, TCFL2 and THADA.	[41]
